# A case of efficacy of bendamustine in heavily pretreated multiple myeloma, refractory to pomalidomide

**DOI:** 10.1002/ccr3.773

**Published:** 2017-03-08

**Authors:** Claudio Cerchione, Davide Nappi, Maria Di Perna, Irene Zacheo, Ilaria Migliaccio, Dalila Salvatore, Marco Picardi, Fabrizio Pane, Lucio Catalano

**Affiliations:** ^1^ HematologyAzienda Ospedaliera Universitaria Federico IIVia Pansini 5Naples80131Italy

**Keywords:** Bendamustine, heavily pretreated, multiple myeloma, pomalidomide, refractory, relapsed

## Abstract

In this report, we would like to highlight the efficacy of bendamustine in a heavily pretreated patient, also refractory to pomalidomide. It is conceivable that different therapy combinations in heavily treated Multiple myeloma (MM) have to be explored, without “a priori” exclusion of ancient drugs, even after failure of the ultimate pharmacological options.

## Introduction

Multiple myeloma (MM) is a clonal malignant disorder derived from an abnormal plasma cell proliferation in the bone marrow that causes anemia, bones lytic lesions and renal injury. If the neoplasm is limited to a restricted area, as bone marrow or soft tissues, it is defined as plasmacytoma.

The natural history of MM has recurrences of active disease defined as relapse when salvage treatment is needed after an off‐therapy period, or refractory disease if nonresponsive during therapy, or progressing within 60 days of last therapy.

In spite of the fact that MM is still an incurable disease, treatment strategies have improved survival in the last decades 1, thanks to the availability of novel agents as proteasome inhibitors (bortezomib, carfilzomib) and immunomodulatory drugs (IMiDs; thalidomide, lenalidomide, and pomalidomide).

In this scenario, the role of many drugs used as salvage therapy after several lines including novel agents in the relapsing/refractory MM is currently undergoing evaluation and, although many new molecules appear promising, their efficacy is still unclear and not always predictable.

Bendamustine is a nitrogen mustard with both purine‐analogue and alkylating agent mechanisms; it is widely used in the treatment of chronic lymphocytic leukemia and low‐grade lymphomas, but is also approved in Europe as single agent and in several combinations with steroid or novel agents (bortezomib, thalidomide, and lenalidomide) for the treatment of relapsed/refractory MM, especially in heavily pretreated patients 2.

In this report, we describe the efficacy of bendamustine combined with bortezomib and dexamethasone (BVD) in a male patient with a long story of relapsed/refractory MM, previously treated with all available drugs, also refractory to pomalidomide, the most recent available IMiDs 3.

## Case Presentation

A 70‐year‐old man was diagnosed with a scantly secretory IgA kappa MM in April 2008, evolved by IgA MGUS recognized 5 years before. At the time of the evaluation, Durie‐Salmon stage was IIIA and ISS was I; PET‐CT showed several areas of intense focal bone involvement. Therapy with thalidomide and dexamethasone (TD) was performed, resulting in a symptomatic stable disease (SD), with persistent positive PET‐CT in the same bone regions plus a new localization on sternum. Second‐line therapy with bortezomib–dexamethasone (VD) for three cycles until March 2009 was performed, but a CT showed heteroplastic tissue wrapping up D2‐D3 vertebra; PET‐CT was positive in the same sites. Third‐line therapy consisting in lenalidomide/dexamethasone (RD) for five cycles + radiotherapy on D2‐D3 was carried out and was followed, in September 2009, by autologous stem cell transplantation after conditioning with high‐dose melphalan (200 mg/m²), and 2 years of interferon (IFN‐*α*2br) maintenance therapy in very good partial response (VGPR). In March 2012, a new relapse in the right shoulder (confirmed by PET‐CT) led further radiotherapy, and the patient remained free of disease for more than 3 years without maintenance. In August 2015, a wide and hard‐consistency lump appeared in the upper‐left quadrant of the abdomen, together with a rising of the Bence‐Jones protein urinary concentration (573 mg/L). Ultrasound scan showed a vascularized mass surrounding and adherent to the small bowel and mesentery. Fine‐needle aspiration cytology (FNAC) was performed on the mass, showing clonal plasma cells and allowing diagnosis of extramedullary plasmocytoma. Consequently, salvage therapy with CED (cyclophosphamide, doxorubicin and dexamethasone) 4 plus radiotherapy on the mass was performed in September 2015, but it was interrupted after the first course due to marrow toxicity (grade III thrombocytopenia). Volume and solidity of the mass were reduced, but Bence‐Jonesprotein increased (progressive disease, PD). Hence, in January 2016, the patient was switched to pomalidomide and dexamethasone, but, after the first course, the disease still progressed.

In February 2016, a seventh‐line salvage treatment was performed employing bendamustine, bortezomib, dexamethasone (BVD: bendamustine 90 mg/sqm IV days 1 and 2, bortezomib 1.3 mg/mq s.c. days 1, 4, 8, 11, dexamethasone 20 mg oral solution/IV days 1, 2, 4, 5, 8, 9, 11, 12 and pegfilgrastim 6 mg s.c. day 4, every 28 days) 5 with the unexpected result of a VGPR, achieved after only one course of treatment, and reduction of more than 90% of urinary K chain concentration and dramatic shrinkage of the abdominal tumor mass. Unfortunately, the patient died before the second course due to progressive cachexia.

## Discussion

Although multiple myeloma is defined as a noncurable disease, nowadays its prognosis is significantly better thanks to a wide spectrum of active drugs. Hence, we often have to face long‐survivor patients receiving several lines of therapy, raising questions on how to combine different treatments and how to manage sequential effects.

Nowadays, available treatments for relapsed/refractory MM are as follows: rechallenge with previously used agents (bortezomib‐ or IMID‐based treatments), with or without autologous bone marrow transplantation, new‐generation IMIDs (pomalidomide), bendamustine‐based treatments, or other alkylating agents. Another option, waiting for new drugs, such as new‐generation proteosome inhibitors (carfilzomib) and monoclonal antibodies (daratumumab and elotuzumab), are combinations of previously used agents, which often demonstrate a synergistic effect also in refractory and heavily pretreated patients.

In this report, we would like to highlight the efficacy of bendamustine in a heavily pretreated patient, also refractory to pomalidomide, the newest available IMiDs. To our knowledge, this sequence of treatment has never been reported before. Moreover, it is relevant the clinical setting where these drugs were administered, as MM finally progressed to massive extramedullary plasmacytoma (intra‐abdominal mass), suggesting that changing in the disease site, hence of disease biology, could have also affected the response to the therapies, particularly active in the lymphoma context 6. It is conceivable that different therapy combinations in heavily treated MM have to be explored, without “a priori” exclusion of ancient drugs, even after failure of the ultimate pharmacological options.

## Authorship

CC: participated in study conception and design, data analysis and interpretation, article drafting and revising it critically for important intellectual content, and gave final approval for publication. He also collected the data. He is responsible for the overall content as guarantor. IM and DS: collaborated in clinical follow‐up of the patient; MDP, DN, MP: participated in study conception and design, data analysis and interpretation and gave final approval for publication; FP and LC: participated in study conception and design, data analysis and interpretation, article drafting and revising it critically for important intellectual content and gave final approval for publication. All authors read and approved the final manuscript.

## Conflict of Interest

None declared.
